# Reviewers for *Journal of Insect Science* (November 2022–October 2023)

**DOI:** 10.1093/jisesa/iead119

**Published:** 2023-12-30

The Editor-in-Chief and the Subject Editors of *Journal of Insect Science* thank the following scientists for their voluntary commitment of valuable professional time and expertise to peer reviewing manuscripts submitted for publication in our journal. The quality and scientific stature of the journal depends on the conscientious efforts of these individuals.

## Top Reviewers (3+ reviews completed)

Athanassiou, Christos

Bartlett, Lewis

Bekelja, Kyle

Bendena, Bill

Bruckner, Selina

Cai, Yao

Chrostek, Ewa

Couvillon, Margaret

Currie, Robert

Daisley, Brendon

Farrokhzadeh, Hadi

Jack, Cameron

Johnson, Reed

Kuś, Krzysztof

Minahan, Danny

Morfin, Nuria

Rassati, Davide

Rinkevich, Frank

Wang, Xianhui

Xia, Xiaofeng

Zhang, Jianzhen

Zong, Shixiang

## Reviewers

Aalto, Anna

Abbasipour, Habib

Adamaki, Christina

Addesso, Karla

Ajayi, Olufemi

Alananbeh, Kholoud M.

Alyokhin, Andrei

Amador-Vargas, Sabrina

Ammar, El-Desouky

Anbalagan, Sankarappan

Anfora, Gianfranco

Anholeto, Luís

Asgari, Sassan

Audisio, Marcela Carina

Aurell, Dan

Avalos, A.

Babu, Arun

Ballal, Chandish

Bandani, Ali

Barredo, Elina

Basu, Priyadarshini

Baumann, Julia

Bernaola, Lina

Bernauer, Olivia

Bonsignore, Carmelo Peter

Brito, Nathália F.

Broadley, Hannah

Brozek, Jolanta

Bujan, Jelena

Butcher, Buntika

C.M., Kalleshwaraswamy

Cameron, Stephen

Cao, Yanghui

Cao, Yu

Capriotti, Natalia

Carcamo, Marcial

Chamberland, Lisa

Chanda, Vikram

Chappell, Thomas

Chaudhury, Muhammad

Chen, Fajun

Chen, Yolanda

Chowdanayaka, Rajanikanth

Cicero, Joseph

Coates, Brad

Cook, Steven

Corby-Harris, Vanessa

Cornara, Daniele

Cornelissen, Bram

Coronado-Rivera, James

Coudron, Thomas

Daane, Kent

Daisley, Brendan

Daniels, Jaret

Davis, Jeffrey

De Meyer, Marc

Deans, Andrew

Del Pozo-Valdivia, Alejandro

Deng, Jian-Yu

Dietrich, Christopher

Dindo, Maria Luisa

Ding, Yike

Dmitriev, Dmitry

do Nascimento, Antonio

Dolezal, Adam

Ebert, Timothy

Ekoja, Edache Ernest

El Sheikha, Aly

Ellis, James

Evangelista, Dominic

Falabella, Patrizia

Faraone, Nicolatta

Favila, Mario

Fell, Richard

Finch, Jonathan

Fine, Julia

Fisher, Adrian

Fladerer, Johannes-Paul

Frizzera, Davide

Fruttero, Leonardo L.

Gang, Wu

Gao, Xi

Geest, Emily

Gerken, Alison

Ghisbain, Guillaume

Ginzel, Matthew

Giraldo, Ysabel

Giray, Tugrul

Gizzi, Alessio

Godoy, Alejandra

Gomez Marco, Francesc

Gordus, Andrew

Gradisek, Anton

Gray, Hannah

Grodowitz, Michael

Gross, Aaron

Grove, Tertia

Hagler, James

Haney, Brian

Hayashida, Rafael

Hayes, Richard

Haziz, Selcuk

He, Hualiang

He, Peng

Hemberger, Jeremy

Heraghty, Sam

Hernandez, Emilio

Hickner, Paul

Hidalgo, Sergio

Hingston, Andrew

Hoffmann, Ary

Holmes, Leslie

Hoover, Shelley

Hou, Chunsheng

Hou, Qiuli

Huang, Zachary

Hubert, Jan

Hulcr, Jiri

Hunter, David

Inouye, David W.

Ioriatti, Claudio

Itoyama, Kyo

Iwan, Dariusz

Jafari, Shahriar

Jetton, Robert

Johnston, Andrew

Juliano, Steven

Jung, Chuleui

Jurenka, Russell

Kacı, Necmiye

Kajtoch, Łukasz

Karban, Richard

Kaya, Harry

Khan, Zahid

Klimes, Petr

Klinger, Ellen

Koch, Jonathan

Kocic, Korana

Kristensen, Michael

Ksentini, Ines

Lara, Ricky

Lau, Pierre

Le, Hanh

Lee, Benjamin

Lee, Yoosook

Leite, Luis

Lesne, Pierre

Letsch, Harald

Li, Bin

Li, Bing

Li, Hou-Feng

Li, Junlan

Li, Yinping

Li, Zhiqiang

Liao, Ling-Hsiu

Li-Byarlay, Hongmei

Lima, Juliana Toledo

Lin, Chia-Hua

Lin, Hao yu

Liu, Tong-Xian

Liu, Wen

Lu, Yujie

Lu, Zhiqiang

Lubin, Yael

Ludwick, Dalton

Luo, Chen

Luo, Yige

MacKell, Sarah

Maistrello, Lara

Maldani, Mohamed

Malé, Pierre-Jean

Maluta, Nathalie Kristine Prado

Mandal, Amit

Manning, Paul

Martinez-Lopez, Oscar

Martini, Xavier

Martins, Nelson

Masetti, Antonio

McKamey, Stuart

McQueen, Eden W.

Meagher, Robert

Mehrabadi, Mohammad

Mikolajewski, Dirk

Miller, Sara

Mishra, Swati

Mitchell, Paula

Mohl, Emily

Mondal, Shoanpius

Moon, Roger

Morgan, Brett

Nagrare, V.

Narain, Ralph

Ndlela, Shepard

Nicodemo, Daniel

Nika, Erifili

Norton, Amie

Oberprieler, Rolf

Oliphant, Zahra

ONeill, Kevin

Ons, Sheila

Pallares, Susana

Papach, Anna

Paulson, Sally

Peach, Dan

Penca, Cory

Peng, Yu

Petrovic, Andjeljko

Pietrykowska-Tudruj, Ewa

Pineda, Samuel

Poche, David

Poh, Karen

Pomeroy, Nelson

Ponti, Luigi

Portilla, Maribel

Pradeep, Appukuttan Nair R.

Prager, Sean

Ranger, Christopher

Rao, Xiang-Jun

Reams, Taylor

Riasat, Tahira

Rihani, Karen

Roda, Amy

Roeder, Diane V.

Ross, Perran

Rossitto De Marchi, Bruno

Roth, Morgan

Rott, Philippe

Rotter, Michael

Sainsbury, James

Saleh, Nicholas

Sánchez-Guillén, Daniel

Sarkar, Surajit

Scannapieco, Alejandra C.

Scharf, Michael

Scieuzo, Carmen

Scott, Jeffrey

Scott, Maxwell

Sedlak, Petr

Sevarika, Milos

Shapiro-Ilan, David

Sharma, Prashant P

Shen, Dongxu

Shepherd, Tonya

Shields, Vonnie

Shimoji, Hiroyuki

Shinoda, Tetsuro

Shrestha, Govinda

Sierra, Ivana

Simmons, Greg

Simon, Chris

Singh, Bashisth

Skevington, Jeffrey

Skourti, Anna

Skuhrovec, Jiri

Smith, Chris

Spivak, Marla

Spodek, Malkie

Srivathsan, Amrita

Stelinski, Lukasz

Stone, Chris

Stouthamer, Richard

Strand, Michael

Stroppa, María Mercedes

Sturrock, Craig J.

Styrsky, John

Swevers, Luc

Sword, Gregory

Tammaru, Toomas

Tarpy, David

Tietjen, Mackenzie

Toyoda, Hideyoshi

Trematerra, Pasquale

Tsagkarakis, Antonios

Uchoa, Manoel

Underwood, Robyn

van Asch, Barbara

Van Eeckhoven, Jens

Verdonckt, Thomas-Wolf

Vesala, Risto

Villegas, Elba

Vogelweith, Fanny

Voigt, Dagmar

Wajnberg, Eric

Wakil FRES, Waqas

Walgenbach, James

Wallingford, Anna

Wallis, Christopher

Wang, Guan-Hong

Wang, Haichuan

Wang, Jialin

Wang, Qiao

Wang, Xingeng

Wang, Yu

Ware, Jessica

Weber, Donald

Wei, Qi

Wei, Shu-Jun

Weintraub, Phyllis

Weissling, Thomas

Werner, Thomas

Whyard, Steve

Wikantyoso, Bramantyo

Will, Torsten

Williams, Geoffrey R.

Williams, III, Livy

Williams, Scott

Wilson, Mikayla

Woller, Derek

Wood, Sarah

Wu, Jianing

Wu, Wei

Xing, Lian Xi

Xu, Shiqing

Xu, Wei

Yan, Fengming

Yang, Xueqing

Ye, Zhengpei

Yee, Wee

Zahoor, Muhammad

Zaviezo, Tania

Zebardast, Nozhat

Zhang, Long-Wa

Zhang, Yong-Jun

Zhang, Yuanmeng

Zhou, Zhongshi

Zhu, Gengping

Zhu, Junwei J.

Zilli, Florencia

